# Doxycycline preserves chondrocyte viability and function in human and calf articular cartilage ex vivo

**DOI:** 10.14814/phy2.14571

**Published:** 2020-09-12

**Authors:** Li Yue, Brian Vuong, Hongwei Yao, Brett D. Owens

**Affiliations:** ^1^ Department of Orthopaedics Warren Alpert Medical School of Brown University and Rhode Island Hospital Providence RI USA; ^2^ Department of Orthopaedic Surgery Stanford University School of Medicine Stanford CA USA; ^3^ Department of Molecular Biology, Cell Biology and Biochemistry Division of Biology and Medicine Brown University Providence RI USA; ^4^ University Orthopedics East Providence RI USA

**Keywords:** apoptosis, chondrocyte viability, doxycycline, glycosaminoglycan, mitochondrial function

## Abstract

Prolonging chondrocyte survival is essential to ensure fresh osteochondral (OC) grafts for treatment of articular cartilage lesions. Doxycycline has been shown to enhance cartilage growth, disrupt terminal differentiation of chondrocytes, and inhibit cartilage matrix degradation. It is unknown whether doxycycline prolongs chondrocyte survival in OC grafts. We hypothesized that doxycycline protects against chondrocyte death and maintains function of articular cartilage. To test this hypothesis, we employed human and calf articular cartilages, and incubated chondrocytes isolated from cartilage or cartilage plugs with doxycycline (0, 1 or 10 μg/ml) at either 37°C or 4°C. Chondrocyte viability, apoptosis, glycosaminoglycan (GAG), collagen, and mechanical test in cartilage plugs were measured. We found that reduced chondrocyte viability, increased chondrocyte apoptosis, reduced GAG contents, and impaired equilibrium modulus in cartilage plugs were observed in a time‐dependent manner at both 37°C and 4°C. Chondrocyte viability was further reduced when the plugs were cultured at 4°C as compared to 37°C. Doxycycline prolonged viability and reduced apoptosis of chondrocytes during culture of cartilage plugs. Functionally, doxycycline protected against reduced production of GAG and collagen II as well as impaired mechanical properties in cartilage plugs during culture. Mechanistically, doxycycline increased mitochondrial respiration in cultured chondrocytes. In conclusion, preservation at 37°C is beneficial for maintaining chondrocyte viability in cartilage plugs compared to 4°C. Incubation of doxycycline protects against chondrocyte apoptosis, reduced extracellular matrix, and impaired mechanical properties in cartilage plugs. The findings provide a potential approach using doxycycline at 37°C to preserve chondrocyte viability in fresh OC grafts for treatment of articular cartilage lesions.

## Graphical abstract

Preservation at 37°C is beneficial for maintaining chondrocyte viability in cartilage plugs compared to 4 degrees Celsius. Incubation of doxycycline protects against chondrocyte apoptosis, reduced extracellular matrix and impaired mechanical properties in cartilage plugs.

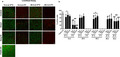

## 1 INTRODUCTION

Surgical transplantation of fresh osteochondral (OC) grafts is an increasingly common procedure used to treat articular cartilage lesions. Although outcomes of fresh OC allograft transplant have been promising in adults, few studies are available in pediatric or adolescent patients (Lyon, Nissen, Liu, & Curtin, 2013; Murphy, Pennock, & Bugbee, 2014). The use of OC allografts doubles between the years 2005 and 2011 (Torrie, Kesler, Elkin, & Gallo, 2015). These OC grafts are usually stored at either −80°C or 4°C until transplantation (Judas, Rosa, Teixeira, Lopes, & Ferreira Mendes, 2007; LaPrade, Botker, Herzog, & Agel, 2009; Ohlendorf, Tomford, & Mankin, 1996; Williams, Dreese, & Chen, 2004). At −80°C, frozen OC allografts have a prolonged maximal storage time and therefore broader graft availability. Under frozen condition, some chemicals are often used to minimize chondrocyte death. However, these chemicals may not be distributed equally across the depth of the graft, which may cause unequal freezing patterns deep into the graft (Judas et al., 2007; Ohlendorf et al., 1996). To avoid this, cartilage allografts are commonly stored at 4°C. Although this storage technique can maintain graft survival for up to 30–40 days, a noticeable decline in chondrocyte viability to levels below 70% occurs after 14 days (Teng, Yuen, & Kim, 2008; Williams et al., 2003; Williams et al., 2005; Williams, Ranawat, Potter, Carter, & Warren, 2007). Thus, storage of OC allografts at 37°C in serum‐free medium has been proposed to preserve chondrocyte viability. Garrity et al. demonstrated that OC allografts from adult canines could maintain chondrocyte viability up to 56 days at 37°C in DMEM medium (Garrity, Stoker, Sims, & Cook, 2012). Preservation at 37°C of canine OC allografts represented a twofold increase in chondrocyte viability over that preserved at 4°C (Garrity et al., 2012; Pallante et al., 2009). The Missouri Osteochondral Allograft Preservation System (MOPS) uses a new solution and specially designed containers that does not require refrigeration. The storage life of bone and cartilage grafts in MOPS is more than twice compared with the preservation method used to preserve OC allografts at room temperature (25°C) by tissue banks. The MOPS allowed preservation of chondrocyte viability for up to 60 days at sufficient levels to result in successful outcomes in a canine model of large femoral condylar articular defects (Cook et al., 2014; Stoker, Stannard, & Cook, 2018). Nevertheless, preservation at 37°C and 25°C poses a risk for susceptibility to infection during the storage.

Use of OC allografts is limited by graft availability due to loss of chondrocyte viability during storage. Chondrocyte viability is essential to maintain the biochemical and biomechanical properties of OC allografts, and this correlates directly to the clinical success of the surgery (Allen et al., 2005; Gross et al., 2008; Malinin, Temple, & Buck, 2006; Williams et al., 2003). These successful outcomes are associated with OC allografts that have at least 70% chondrocyte viability at 28–60 days after procurement (Cook et al., 2014). Current standards at tissue banks require microbiological and serological safety testing of graft specimens that typically lasts up to 14 days. The short practical shelf life of 2 weeks has narrowed the time window for allograft implantation to merely 15–28 days (Familiari et al., 2018). Therefore, the ability to maintain chondrocyte viability and preserve function of fresh OC grafts from young donors is a critical clinical need.

Doxycycline is a widely available, inexpensive, and well‐tolerated antibiotic that is used to treat bacterial infections. Matrix metalloproteinases (MMPs) are the main extracellular matrix enzymes for collagen degradation, which are upregulated after cartilage injury or in arthritis (Brandt et al., 2005; Shlopov, Stuart, Gumanovskaya, & Hasty, 2001). Oral administration of doxycycline reduced MMP‐13 activity and improved tendon‐to‐bone healing after rotator cuff repairs in rats (Bedi et al., 2010). Doxycycline also stimulated cartilage growth and disrupted the terminal chondrocyte differentiation (Cole et al., 1994). In another study, doxycycline treatment augmented chondrogenesis of human bone marrow‐derived mesenchymal stem cells in vitro, and inhibited MMP‐13 expression in pellet cultures and within rat osteochondral defects (Lee, O'Malley, Friel, & Chu, 2013). These results suggest the potential use of doxycycline to improve cartilage repair to delay the onset of osteoarthritis. It is not clear whether doxycycline maintains chondrocyte survival and OC graft function during storage. Therefore, we hypothesized that doxycycline prolongs chondrocyte viability and protects function of OC grafts ex vivo. To test this hypothesis, we employed knee articular cartilage from young human donors and adolescent bovines, and determined the optimal concentrations of doxycycline for maintaining chondrocyte viability in OC grafts.

## 2 MATERIALS AND METHODS

### 2.1 Isolation and treatment of human cartilage chondrocytes

Expired human OC grafts from 10 healthy male donors (ages between 17 and 25 years old, mean age is 24 years) were obtained from the Musculoskeletal Transplant Foundation (MTF) for research use only. The OC grafts are used for experiments around 2 months after OC graft collection. Articular cartilage was sliced into small pieces in a 15 cm Petri dish, then transferred into 50 ml Falcon centrifuge tube. Pronase was added with 2 mg/ml for digestion for 30 min at 37°C with shaking. Pronase solution was removed, and small cartilage pieces were washed with DMEM/F‐12 and centrifuged at 1,500 rpm for 5 min. After DMEM/F‐12 was removed from the tube, digested tissue pieces were incubated with 1 mg/ml of collagenase from Clostridium histolyticum (type IA) in HBSS for 6–8 hr at 37°C under shaking. Enzymatic reaction was arrested by adding DMEM/F‐12 completed medium, containing 10% FBS and 1% penicillin–streptomycin. The digested solution was filtered with 70 µm cell strainer to get rid of the clumps. Filtered solution was centrifuged at 1,500 rpm for 5 min. After removing the supernatant, cell pellets were resuspended in DMEM/F‐12 completed medium and seeded in 10 cm cell culture dishes. Primary cells were maintained at 37°C with 5% CO_2_ and grown until 80%–90% confluency before passaging. Cells with passage 3 were cultured in six‐well plates or Falcon^®^ four‐well chambered cell culture slides (Corning Incorporated, Big Flats, NY). When reaching 80%–90% confluency, cells were treated with or without doxycycline (1 and 10 μg/ml) until 14 days. Images of cellular morphology were taken by Nikon Eclipse TS100 inverted microscope (Avon, MA, USA) at different time points.

### 2.2 Culture and treatment of calf cartilage plug

Femoral condyle articular cartilage was aseptically harvested within 4 hr of death from bovines (seven male calves, 50–70 lbs of body weight), which were purchased from a local slaughter. The cartilage was punched into plugs with a diameter of 4 mm. Cartilage plugs on the day of calf sacrifice were used and referred to as the Fresh d0 group. The plugs were cultured in 48‐well plates at 37°C or 4°C in the presence or absence of doxycycline (1 and 10 μg/ml) until day 63. The DMEM medium containing MEM nonessential amino acids solution, L‐ascorbic acid, selenium, insulin, transferrin, and sodium pyruvate, was changed twice a week. The protocol for processing calf cartilage was approved by the IRB committee at the Rhode Island Hospital.

### 2.3 LIVE/DEAD assay

Chondrocyte viability in cartilage plugs was assessed using LIVE/DEAD™ Viability/Cytotoxicity Kit. This assay detects intracellular esterase activity in live cells with green‐fluorescent calcein‐AM and loss of plasma membrane integrity in dead cells with red‐fluorescent ethidium homodimer‐1. Staining was visualized under confocal microscopy. Confocal images were acquired with a Nikon C1si confocal (Nikon Inc., Mellville, NY) using diode lasers 488 and 561. Serial optical sections were performed with EZ‐C1 computer software (Nikon Inc., Mellville, NY). Z serial sections were collected at 0.5 µm with a 20× Plan Apo lens. Three fields per sample were examined with each channel acquired separately by invoking frame lambda. Samples ranged in thickness between 20 and 100 µm. For each confocal Z stack, approximately 10 µm were cropped from the bottom of the stack prior to the final projection to eliminate frames that showed damage to the core due to handling. Projected RGB images were analysed in ImageJ (National Institutes of Health, Springfield VA). A size criterion of greater than 10 µm^2^ was used for both live and dead cells. Each image was channel split, and thresholded for total cell counts. To facilitate accurate counts, the median and watershed filters were used. Chondrocyte viability was calculated as the percentage of live cells relative to the total number of cells.

### 2.4 Glycosaminoglycan (GAG) assay

Cartilage plugs were removed from their respective mediums and their wet weights were measured immediately. The plugs were then lyophilized for 6 hr in a freeze‐dry machine. After recording the dry weights of these freeze‐dried plugs, the samples were digested overnight in papain working solution (10 ml PBE/cysteine, 50 μl papain enzyme). More specifically, each plug was digested in 1 ml of papain working solution for 12 hr at 60°C. The PBE/cysteine solution was prepared from 120 ml of PBE buffer (7.1 g Na_2_HPO_4_, 1.86 g Na_2_EDTA·2H_2_O, 500 ml dH_2_O, pH 6.5). A glycosaminoglycan‐dimethylmethylene blue (GAG‐DMMB) assay was used to assess GAG content in the digested cartilage plugs. In this assay, GAG levels were quantified via absorbance measurements, specifically the turbidity resulting from the formation of GAG‐DMMB complexes. Results were normalized to a standard curve generated by different concentrations of chondroitin sulfate diluted in papain working solution.

### 2.5 Mechanical test

To assess the mechanical properties of cartilage plugs, compressive testing was performed using an ElectroPuls^TM^ E1000 dynamic test instrument (Instron, Norwood, MA) at room temperature. The compressive testing sequence consisted of: (a) Creep −2 gf (0.02 N = 2 gf) tare load to ensure contact between the actuator and explant sample; (b) Stress relaxation at −10% strain for 30 min (until equilibrium); (c) Unconfined dynamic compression by superimposing the aforementioned −10% equilibrium strain with a 1% sinusoidal strain (fluctuation of strain between −11% and −9%) at a frequency of 1 Hz for 45 cycles. The load exerted by the cartilage explant in reaction to static and dynamic compression sequences was measured by a 10 N load cell. Strain applied was measured using a linear variable differential transformer sensor in contact with a platform attached to and at the same level as the compression actuator. Position, digital position, load (reaction force), and strain (displacement) were recorded in real time at a sampling frequency of 10 Hz for both compression sequences through WaveMatrix software (Instron, Norwood, MA). Raw data recorded by WaveMatrix was then processed through MATLAB (MathWorks, Natick, MA). Equilibrium modulus was calculated using stress at *t* = 30 min during stress relaxation. Dynamic modulus was calculated using the average of minimum and maximum stresses at the 20th, 30th, and 40th cycle of cyclic loading.

### 2.6 Histological processing and immunostaining capture

Calf cartilage plugs treated with or without doxycycline were harvested at day 14, day 28, day 48, day 56, and day 63. The plugs were fixed in 10% buffered formalin for 3 days and dehydrated in 70% ethanol followed by paraffin embedding. Paraffin‐embedded plugs were cut into 4‐μm histologic sections and mounted on glass slides. The sections were deparaffinized with three changes of xylene for 5 min each, and then rehydrated in an ethanol series (100%, 95%, twice, 5 min each and 70%, 5 min) and finally in distilled water for 5 min. Immunostaining was performed for at least three plugs in each group. For all the immunostaining, three separate fields per stain were imaged under microscope. RGB images were acquired with a Nikon E800 microscope (Nikon Inc., Mellville, NY) using a 20× Plan Apo objective and a RT3 digital camera (Diagnostic Instruments, Sterling Heights, MI).

### 2.7 Safranin O/fast green staining

Safranin O (red) stains proteoglycans, fast green (green) stains background, and hematoxylin stains nuclei in cartilages. The cartilage is stained orange to red, and the nuclei are stained black. In brief, deparaffinized samples were hydrated, then the slides were immersed in Weigert's hematoxylin for 5 min, followed by rinsing with distilled water for 2 min until excess stops leaching out of tissue. The sections were dipped once in 1% acid alcohol for 2 s followed by gently rinsing in distill water for 2 min. The sections were then further stained in 0.02% fast green solution for 30 s, and dipped in 1% acetic acid for 30 s and excess liquid was absorbed using paper towel. Subsequently, the sections were stained with Safranin O solution for 10 min followed by rinsing in 95% ethanol. After dehydrating with three changes of 95% ethanol, two changes of 100% ethanol, three changes of xylene (1 min each), added 1–3 drops of Cytoseal 60 mounting medium and a cover slip was placed over the stained tissue.

### 2.8 Hematoxylin and eosin (H&E) staining

After deparaffinization and rehydration of tissue sections through xylenes and graded alcohol series, the sections were stained in Mayer's Hematoxylin solution from NovaUltra^TM^ H&E stain kit (IHC World LLC, Woodstock, MD) for 2 min flowed by rinsing in running tap water for 2 min. Then the slides were rinsed in 95% ethanol for 30 s, subsequently, the sections were counterstained in eosin solution for 45 s followed by quickly rinsing in 95% ethanol for 10 dips. Finally, the sections were dehydrated and mounted with coverslips.

### 2.9 Apoptosis

After deparaffinizing and rehydrating tissue sections, the ApopTag^®^ Peroxidase In Situ Apoptosis Detection Kit (EMD Millipore, Temecula, CA) was used to detect apoptotic cells in situ by labeling and detecting DNA strand breaks using the TUNEL method. The deparaffinized tissue sections were incubated with freshly diluted proteinase K for 15 min at room temperature followed by rinsing with distill water twice, for 2 min each. Endogenous peroxidase is quenched in 3.0% hydrogen peroxide in PBS for 5 min at room temperature followed by rinsing twice in PBS for 5 min each. Excess liquid was gently taped off and carefully aspirated around the section, then 75 µl/5 cm^2^ of equilibration buffer was immediately applied directly on the specimen. The sections were incubated for at least 10 s at room temperature. After excess liquid was gently taped off and carefully aspirated around the section, immediately 55 µl/5 cm^2^ of working strength TdT enzyme was pipetted onto the section and the slides were incubated in a humidified chamber at 37°C for 1 hr. Then sections were agitated for 15 s and incubated with working strength stop/wash buffer followed by washing with three times with PBS, 1 min each. Excess liquid was gently taped off and carefully aspirated around the section, and 65 µl/5 cm^2^ of anti‐digoxigenin conjugates were applied to the slides to incubate in a humidified chamber for 30 min at room temperature. After washing four changes of PBS for 2 min, 75 µl/5 cm^2^ of peroxidase substrate was applied to cover the specimens for 3 min followed by washing with three times of distill water, 1 min each, and incubating in distill water for 5 min. After counterstaining in 0.5% methyl green solution for 10 min at room temperature, the sections were washed in three changes of distill water and three changes of 100% N‐butanol, dipping the slides 10 times each in the first and second washes, followed by 30 s without agitation in the third wash. Finally, the sections were dehydrated and mounted with coverslips.

### 2.10 Measurement of mitochondrial oxidative phosphorylation

The Seahorse XF Cell Mito Stress Test is used to measure mitochondrial respiration in human chondrocytes. This test measures key parameters of mitochondrial oxidative phosphorylation by directly evaluating the oxygen consumption rate (OCR) of the cells. After optimization, we seeded cells in Seahorse XF24 microplates (Seahorse Bioscience, Billerica, MA) overnight prior to the measurement of OCR. After optimization, we seeded human chondrocytes (60,000 cells/well) in a Seahorse XF24 microplate (Seahorse Bioscience, Billerica, MA) overnight prior to the measurement of OCR as described earlier (Yao et al., 2019). The culture media were changed into the assay medium, which was the XF base medium supplemented with 1 mmol/L pyruvate, 2 mmol/L of glutamine, and 10 mmol/L glucose. Cells were incubated with assay medium for 45 min–1 hr before the assay in a non‐CO_2_ incubator at 37°C. Injections of oligomycin (1 μmol/L final), carbonyl cyanide p‐trifluoromethoxyphenylhydrazone (50 mmol/L final), and rotenone/antimycin A (0.5 μmol/L, final) were diluted in the assay media, and loaded onto ports A, B, and C of prehydrated Seahorse XF Sensor Cartridge (Seahorse Bioscience, Billerica, MA), respectively. The Seahorse XF Analyzer was calibrated and the assay was performed using the Seahorse XF Cell Mito Stress Test Assay protocol as suggested by the manufacturer (Pike Winer & Wu, 2014). The OCR in living cells was detected by the Seahorse XF24 Analyzer (Seahorse Bioscience, Billerica, MA). Number of live cells in representative wells were counted before Seahorse assay, and these cell counts were used to normalize OCR.

### 2.11 Immunofluorescence for collagen II

Human chondrocytes with passage 3 were cultured at a density of 3.3 × 10^4^ cells/cm^2^ in Falcon^®^ 4‐well Chambered cell culture slides. Cells were stained for collagen II by immunofluorescence once they reached ~90% confluence. Chondrocytes were fixed with 10% formalin for 10 min after washing with PBS three times. Fixed cells were incubated for 10 min with PBS containing 0.1% Triton to increase the penetration of the antibody. After washing with PBS three times for 5 min each, cells then were incubated with 10% normal goat serum in PBST (PBS + 0.1% Tween 20) for 30 min to block unspecific binding of the antibodies. Cells were incubated in the diluted anti‐collagen II antibody (1:1,000 dilution, ab34712, Abcam, Cambridge, MA) with 10% normal goat serum in PBST in a humidified chamber overnight at 4°C. Cells were then incubated with goat anti‐rabbit IgG H&L (Alexa Fluor^®^ 594) (1:1,000 dilution, ab150080, Abcam, Cambridge, MA) in 10% normal goat serum in PBST BSA for 1 hr at room temperature in dark. After washing three times with PBS for 5 min each in the dark, the slides were mounted using VECTASHIELD Hardset mounting medium with DAPI (Vector Laboratories Inc., Burlingame, CA). Three separate fields per stain were imaged under Nikon's Eclipse E800 fluorescence microscope (Avon, MA). The images were captured at 20× magnification.

### 2.12 Statistical analysis

All statistical analyses were performed using GraphPad PRISM 8 software. Results were presented as mean ± standard error of the mean (*SEM*) of different samples, and considered significant when *p* < .05. Each experiment was performed at least three times. Data were examined by one‐way ANOVA with Bonferroni post hoc multiple comparison to measure the effect of one factor, or by the two‐way ANOVA with Bonferroni post hoc multiple comparisons to measure the effects of two factors. A *t*‐test was used to compare any two groups.

## 3 RESULTS

### 3.1 Human chondrocytes from knee articular cartilage were successfully isolated and cultured

**FIGURE 1:**
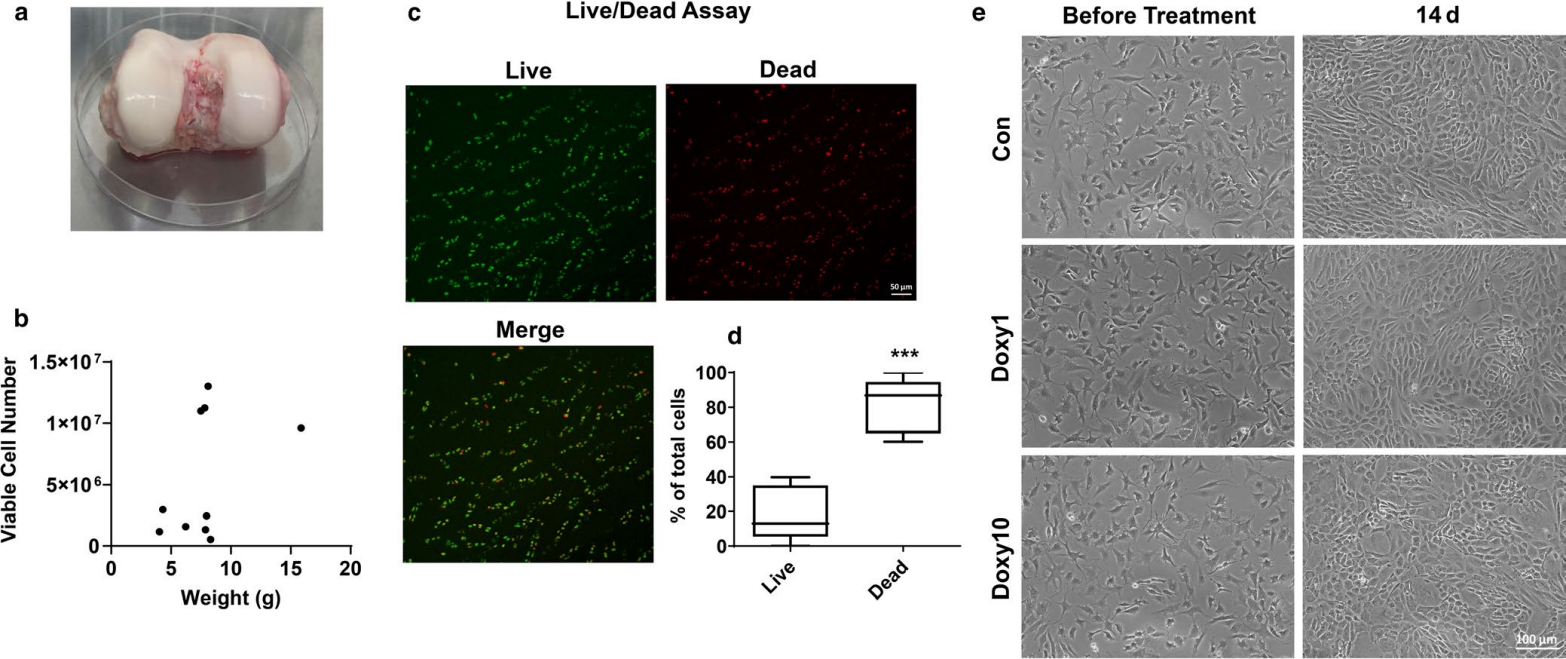
Human chondrocytes from OC graft cartilage were successfully cultured. Expired human OC grafts from 10 healthy donors from MTF for research use only. Human cartilage was punched into the plugs with a diameter of 4 mm followed by LIVE/DEAD assay. LIVE/DEAD assay detects intracellular esterase activity in live cells with green‐fluorescent calcein‐AM staining and loss of plasma membrane integrity in dead cells with red‐fluorescent ethidium homodimer‐1 staining. Cartilage was sliced into small pieces and digested with 1 mg/ml of collagenase in HBSS for 6–8 hr at 37°C followed by digestion with 2 mg/ml of pronase. The digested solution was filtered with 70 µm cell strainer and centrifuged. Then cell pellets were resuspended in DMEM/F‐12 completed medium and seeded. When passage 3 human cells reached 80%–90% confluency in six‐well plates, cells were treated with or without doxycycline (1 and 10 μg/ml) until 14 days. (a) A representative image of one expired OC graft. (b) Viable cell number and sliced human cartilage weight from MTF osteochondral graft (*N* = 10). (c) Representative images of Live/Dead staining for human cartilage chondrocytes (*N* = 6). Live cells were labeled in green fluorescence and dead cells in red fluorescence. (d) Quantification of the percentage of Live and Dead cells using ImageJ software (*N* = 6). (e) Morphology of cultured chondrocytes before treatment and on 14‐day treatment (*N* = 6)

Chondrocytes were isolated from expired human knee articular cartilage (Figure 1a). The weights of human cartilage grafts from 10 donors were measured. After digestion and isolation, the number of viable chondrocytes was counted. Human cartilage weight and viable cell number varied within these 10 donors (Figure 1b). Human cartilage was punched into the plugs with a diameter of 4 mm followed by LIVE/DEAD assay. Mean percentage of live chondrocytes from expired OC grafts was 18.59%, while dead chondrocytes was 81.41%. We also found that the number of live and dead cells varied within these 10 donors (Figure 1c and d). At passage 3, chondrocytes were cultured in six‐well plates in DMEM/F‐12 completed medium, containing 10% FBS and 1% penicillin‐streptomycin (10,000 U/ml) at 37°C, and 5% CO_2_ in a humidified incubator. Cells were incubated with 1 μg/ml and 10 μg/ml of doxycycline for 14 days. Cell morphology was observed under microscopy. Cultured cells stopped growing when they reached confluence (contact inhibition). These cells made a progressive change into a spindle‐shaped morphology during culture (Figure 1e). There was no significant difference on chondrocyte morphology between the control group and doxycycline‐incubated groups (1 μg/ml and 10 μg/ml) at 14 days (Figure 1e). These results suggest that chondrocytes are successfully isolated from human articular cartilage, and doxycycline has no effect on their morphology.

### 3.2 Doxycycline increased collagen II in cultured human chondrocytes

**FIGURE 2:**
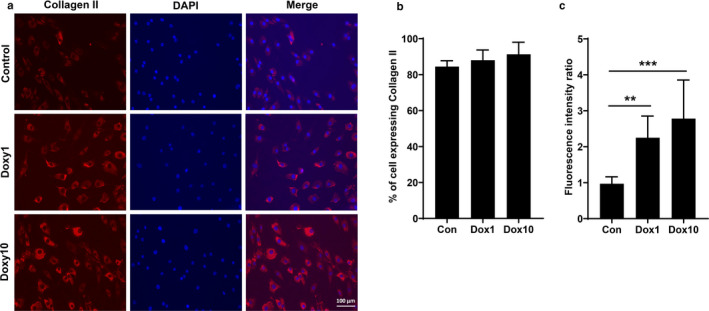
Doxycycline augmented collagen II expression in cultured human chondrocytes at 14 days. When passage 3 human cells from MTF OC grafts reached 80%–90% confluency in 4‐well chambered cell culture slides, cells were treated with or without doxycycline (1 and 10 μg/ml) until 14 days. (a) Fluorescence microscope images of immunostained collagen II (red). Nuclei were counterstained with DAPI (blue). (b) Quantification of the percentage of the cells expressing collagen II at 14 days using ImageJ software. (c) Quantification of fluorescence intensity of collagen II at 14 days using ImageJ software. Data are expressed as mean ± *SEM* (*N* = 6). **p < .01 and ***p < .001 for comparison of the two groups

Normally healthy articular cartilage express type II collagen but not type I collagen. Thus, we determined whether doxycycline alters collagen II expression in cultured chondrocytes. Chondrocytes were isolated from human cartilage, and treated with or without 1 μg/ml or 10 μg/ml of doxycycline for 14 days at 37°C in vitro. Expression of collagen II was measured by immunofluorescence. Cultured cells expressed high levels of collagen II in cytoplasm (red), with DAPI staining in nucleus (blue) (Figure 2a). Number of cells expressing collagen II was not significantly altered by doxycycline (84.46% in control, 88.03% in 1 μg/ml of doxycycline, and 91.32% in 10 μg/ml of doxycycline) (Figure 2b). Incubation with doxycycline (1 μg/ml and 10 μg/ml) significantly augmented the fluorescence intensity of collagen II in human chondrocytes (Figure 2c). Altogether, these results indicate that doxycycline increases collagen II synthesis in cultured human chondrocytes in a subpopulation‐specific manner.

### 3.3 Doxycycline protects against reduced chondrocyte viability in calf cartilage plugs during culture

**FIGURE 3:**
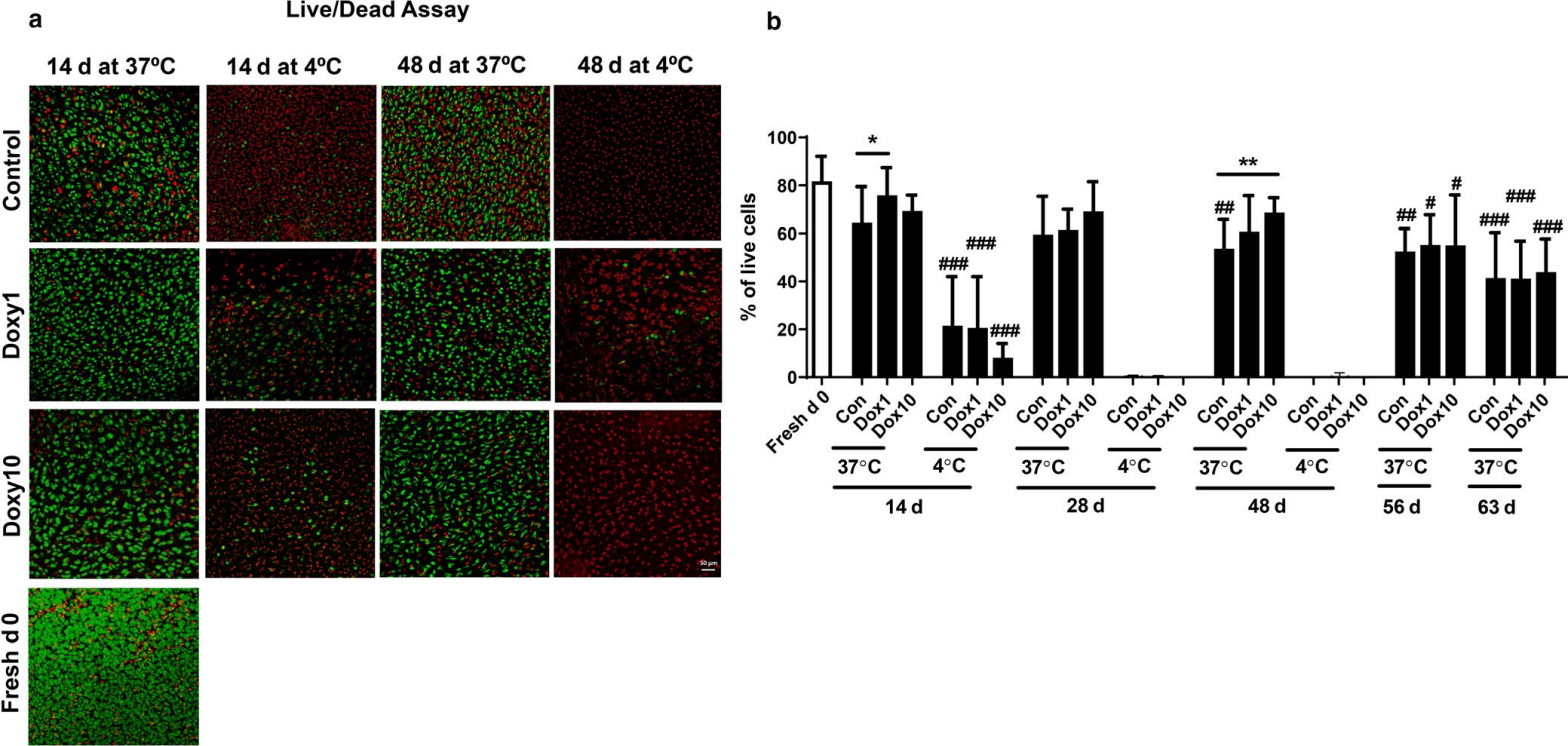
Doxycycline protected against reduction of chondrocyte viability in calf cartilage plugs during culture. Calf knee cartilage was punched into plugs with a diameter of 4 mm. The cartilage plugs obtained on the day of slaughter were used as the Fresh d0 group. The plugs were cultured in 48‐well plates at 37°C or 4°C in the presence or absence of doxycycline (1 μg/ml and 10 μg/ml) until 63 days. Chondrocyte viability in plugs was examined using the LIVE/DEAD Viability/Cytotoxicity kit under confocal microscopy. (a) Representative images of Live/Dead staining on days 0, 14, and 48 at 37°C and 4°C (*N* = 5–7). Green fluorescence indicates the live cells, and red fluorescence indicates the dead cells. (b) Quantification of Live/Dead staining using ImageJ software (*N* = 5–7). ^#^ *p* < .05, ^##^ *p* < .01, ^###^ *p* < .001 versus Fresh d0. *p < .05 and **p < .01 for comparison of two groups

Due to insufficient human cartilages, we employed fresh calf knee cartilage for further study on chondrocyte by doxycycline. We first compared the effect of culture temperature on chondrocyte viability in cartilage plugs. When cartilage plugs were cultured at 37°C, the chondrocyte viability in the control group was 81.72%, 64.49%, 59.54%, 53.64%, 52.42%, and 41.46% at day 0, 14, 28, 48, 56, and 63, respectively. The viability of chondrocytes was 21.58%, 0.11%, and 0% when the plugs of the control group were cultured at 4°C for 14, 28, and 48 days, respectively (Figure 3a and b). This suggests that chondrocyte viability decreases during culture, and culture at 37°C is beneficial for chondrocyte viability compared to culture at 4°C.

Doxycycline incubation caused a significant increase in the viability of chondrocytes, which was demonstrated by an increase of 17.8% on day 14 (1 μg/ml of doxycycline vs. control) and 28.2% on day 48 (10 μg/ml of doxycycline vs. control) in cartilage plugs at 37°C (Figure 3a and b). Chondrocyte viability at 4°C was not significantly different between control and doxycycline‐treated groups. At each time point, no significant differences in chondrocyte viability were observed between 1 μg/ml and 10 μg/ml of doxycycline at both 37°C and 4°C (Figure 3a and b). These results suggest that doxycycline incubation protects against reduction of chondrocyte viability in cartilage plugs at 37°C.

### 3.4 Doxycycline protected against chondrocyte loss in calf cartilage plugs during culture

**FIGURE 4:**
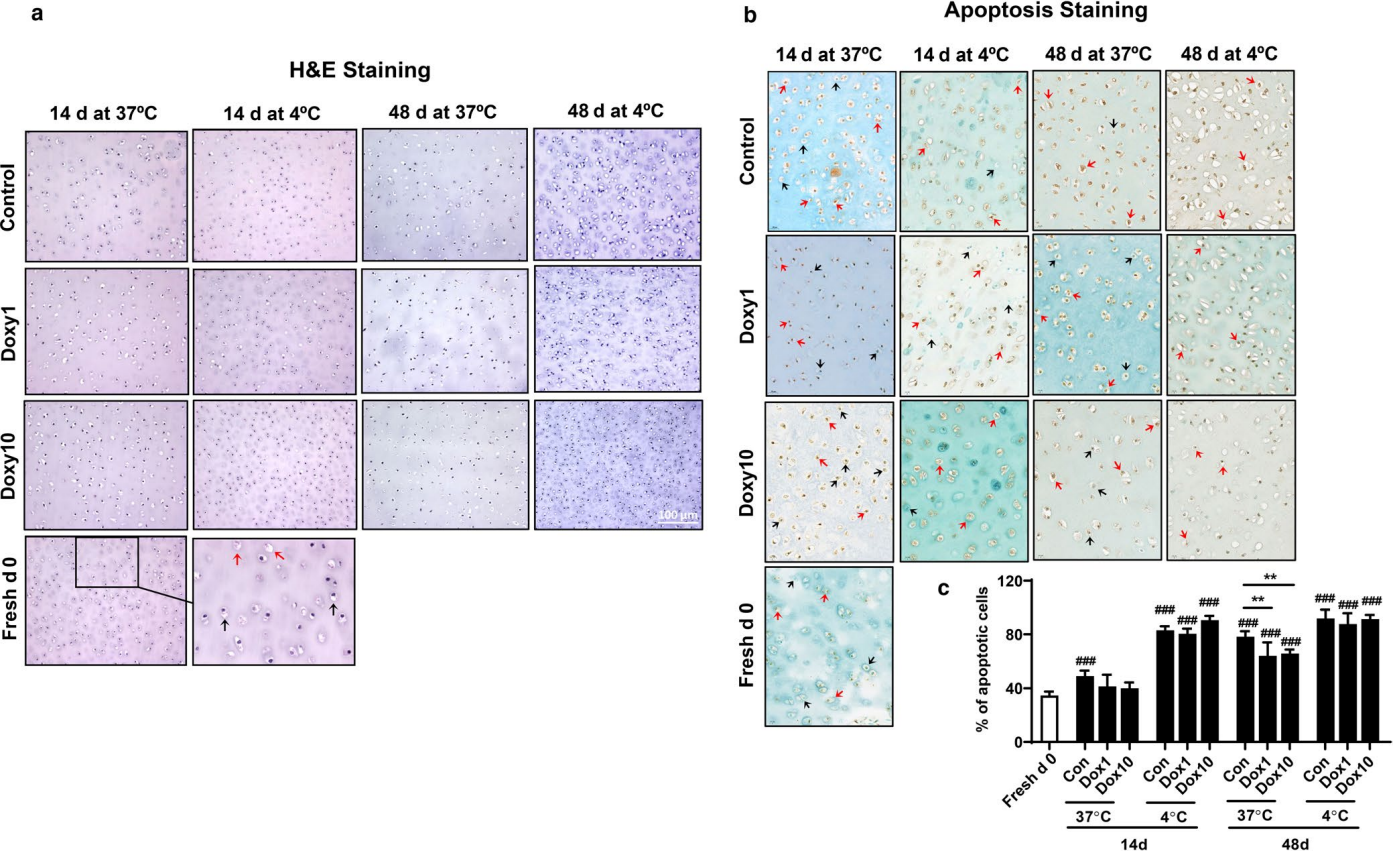
Doxycycline ameliorated chondrocyte loss and apoptosis in calf cartilage plugs during culture. Calf knee cartilage was punched into plugs with a diameter of 4 mm. The cartilage plugs obtained on the day of slaughter were used as the Fresh d0 group. The plugs were cultured in 48‐well plates at 37°C or 4°C in the presence or absence of doxycycline (1 μg/ml and 10 μg/ml) until 48 days. The plugs were fixed in 10% buffered formalin for 3 days and dehydrated in 70% ethanol, and immediately processed for paraffin embedding. Paraffin‐embedded plugs were cut into 4‐μm histologic sections and mounted on glass slides. Formalin‐fixed, paraffin‐embedded sections were stained with hematoxylin and eosin (H&E) and ApopTag^®^ peroxidase using in situ apoptosis detection kit. (a) Representative images of 14‐day and 48‐day H&E staining (*N* = 6). Black arrows indicate classical chondrocytes. Red arrows indicate unhealthy cells. (b) Representative images of 14‐day and 48‐day apoptosis staining (*N* = 6). Black arrows indicate nonapoptotic cells. Red arrows indicate apoptotic cells. (c) Quantification of apoptosis staining using ImageJ software (*N* = 6). ^###^ *p* < .001 versus Fresh d0. **p < .01 for comparison of two groups

In H&E stained sections, classical chondrocyte has a round, pale‐stained cytoplasm and a small, dark blue hyperchromatic nucleus. Each chondrocyte has one round to ellipsoid nucleus and pale‐stained cytoplasm. As shown in Figure 4a, nuclei staining was gradually lost, and increased empty lacunae in cytoplasm was observed in the control group and doxycycline‐treated group when cartilage plugs were cultured for 14 and 48 days compared to day 0. Empty lacunae and absent nuclei staining were further increased at 4°C as compared to 37°C of the culture at the corresponding time points in both the control and doxycycline‐treated groups. However, more empty lacunae and absent nuclei staining were observed in the control group as compared to doxycycline‐treated group at 4°C for 48 days culture (Figure 4a).

### 3.5 Doxycycline ameliorated apoptotic chondrocytes in calf cartilage plugs during culture

We determined whether the protective role of doxycycline against chondrocyte loss during culture is associated with reduced apoptosis. Apoptosis was detected using the ApopTag^®^ Peroxidase In Situ Apoptosis Detection Kit. Apoptosis in chondrocytes was gradually increased when cartilage plugs were cultured for 14 days and 48 days (Figure 4b and c). Furthermore, increased apoptosis was observed at 4°C as compared to those at 37°C at the corresponding time points (Figure 4b and c). Apoptotic chondrocytes were reduced when cartilage plugs were incubated with doxycycline (1 μg/ml and 10 μg/ml) on 48 days at 37°C (Figure 4b and c). These results suggest that doxycycline incubation reduces apoptosis in chondrocytes when cartilage plugs are cultured for 48 days at 37°C.

### 3.6 Doxycycline increased mitochondrial respiration in cultured human chondrocytes

**FIGURE 5:**
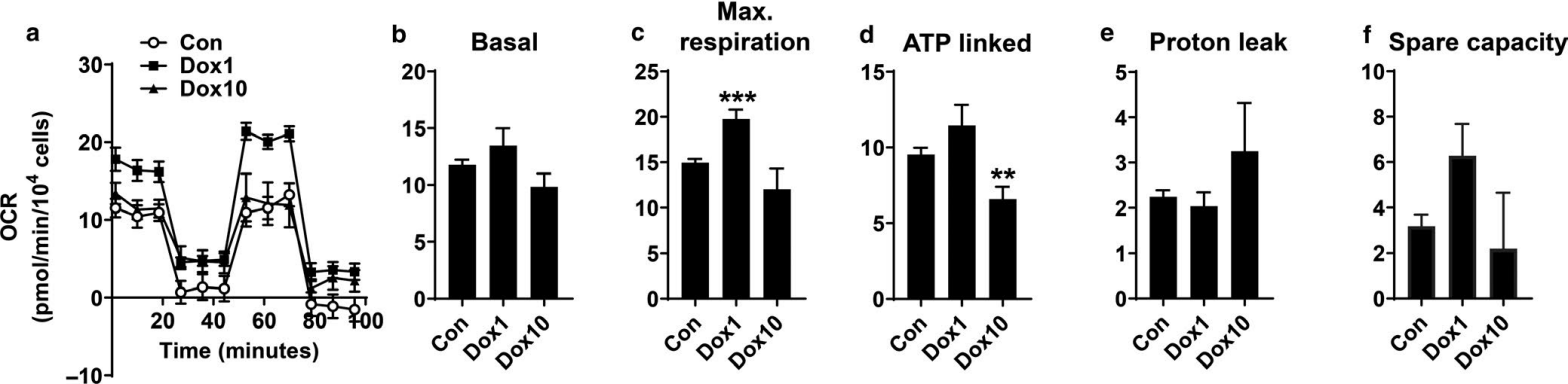
Doxycycline increased mitochondrial respiration in cultured chondrocytes isolated from human cartilage. Calf knee cartilage was punched into plugs with a diameter of 4 mm. The plugs were cultured in 48‐well plates at 37°C or 4°C in the presence or absence of doxycycline (1 μg/ml and 10 μg/ml) for 14 days. Then the human chondrocytes were removed from culture plates and seeded at a density of 60,000 cells/well in Seahorse XF24 microplates overnight prior to the measurement of OCR. The culture media were changed to the assay medium and cells were incubated with the assay medium for 45 min–1 hr before the assay in a non‐CO_2_ incubator at 37°C. Injections of oligomycin (1 μmol/L final), carbonyl cyanide p‐trifluoromethoxyphenylhydrazone (50 mmol/L final), and rotenone/antimycin A (0.5 μmol/L final) were diluted in the assay medium, and loaded onto ports a, b, and c of prehydrated Seahorse XF Sensor Cartridge, respectively. The Seahorse XF Analyzer was calibrated and the assay was performed using the Seahorse XF Cell Mito Stress Test Assay protocol. The OCR in living cells was detected by the Seahorse XF24 Analyzer. Numbers of living cells in representative wells were counted before Seahorse assay, and these cell counts were used to normalize OCR. (a) Oxygen consumption rate (OCR) of cultured cells for vehicle control group (Con), 1 μg/ml of doxycycline‐treated group, and 10 μg/ml of doxycycline‐treated group (*N* = 6). (b) Basal respiration (c) Maximal respiration (d) ATP‐linked respiration (e) proton leak (f) Spare capacity (*N* = 6). **p < .01 and ***p < .001 versus control group

Mitochondrial function plays an important role in modulating viability and apoptosis in chondrocytes (Chang, Huo, Li, Wu, & Zhang, 2015; Collins et al., 2016; Koike et al., 2015). Thus, we measured mitochondrial oxidative phosphorylation in chondrocytes with or without doxycycline treatment using a Seahorse XF Analyzer. We found that treatment with 1 µg/ml of doxycycline at 37°C for 14 days increased mitochondrial respiration as reflected by augmented maximal respiration and spare capacity. The levels of basal respiration, ATP‐linked respiration or proton leak were not altered by 1 µg/ml of doxycycline treatment, whereas 10 μg/ml of doxycycline decreased the level of ATP‐linked respiration. (Figure 5a–f). These results suggest that 1 µg/ml of doxycycline increases mitochondrial respiration in chondrocytes at 37°C.

### 3.7 Doxycycline protected against GAG reduction in calf cartilage plugs during culture

**FIGURE 6:**
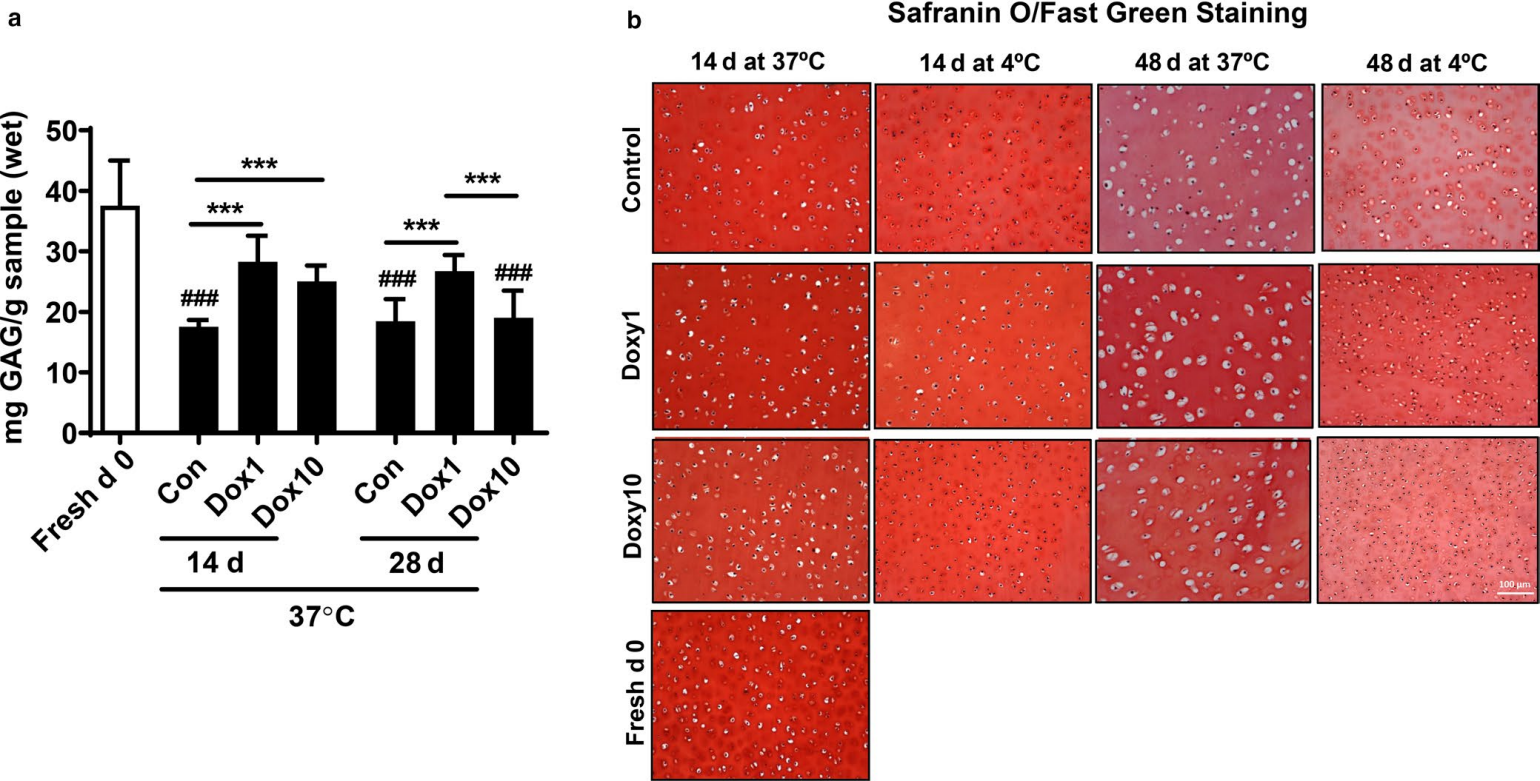
Doxycycline protected against GAG reduction in calf cartilage plugs during culture. Calf knee cartilage was punched into plugs with a diameter of 4 mm. The cartilage plugs at the day of slaughter were used as Fresh d0 group. The plugs were cultured in 48‐well plates at 37°C or 4°C in the presence or absence of doxycycline (1 μg/ml and 10 μg/ml) for different time points. The plugs were harvested at different time points. The GAG‐DMMB assay was used to assess GAG content in the digested cartilage plugs. Some plugs are fixed in 10% buffered formalin for 3 days and dehydrated in 70% ethanol, then immediately processed for paraffin embedding. Paraffin‐embedded plugs were cut into 4‐μm histologic sections and mounted on glass slides. Formalin‐fixed, paraffin‐embedded sections were stained with Safranin O/Fast Green. (a) GAG‐DMMB assay results presented as average GAG ± *SEM* (*N* = 6). ^###^ *p* < .001 versus Fresh d0. ***p < .001 for comparison of two groups. (b) Representative images of 14‐day and 48‐day Safranin O/Fast Green staining (*N* = 6)

Compressive stiffness of cartilage is mainly determined by GAG and water content, while tensile strength and other short‐term dynamic properties are primarily affected by collagen content. We, therefore, measured the content of GAG by the GAG‐DMMB assay. We found that that at day 0, GAG content in the cartilage plugs was 37.54 ± 7.5 mg GAG/g (wet weight), which was significantly reduced at both days 14 and 28 in culture (Figure 6a). Incubation with doxycycline at 1 and 10 µg/ml significantly increased the contents of GAG at day 14 compared to the control group (Figure 6a). At day 28, doxycycline at a concentration of 1 µg/ml, but not 10 µg/ml, significantly augmented GAG contents when compared with the control group (Figure 6a).

The intensity of Safranin O staining is proportional to the proteoglycan content, including GAG, in the cartilage tissue. Safranin O binds to GAG and shows an orange to red color, and the nuclei are stained black. In order to confirm the effect of doxycycline on GAG loss, formalin‐fixed, paraffin sections from individual cartilage plugs were stained with Safranin O for indicating GAG and fast green for indicating nonproteoglycan background. As the culture time extended, GAG content was decreased at both 37°C and 4°C. Doxycycline at 1 μg/ml protected against GAG reduction as compared to the 10 μg/ml doxycycline concentration and control groups (Figure 6b). More GAG loss was observed at 4°C than that in the same group at 37°C (Figure 6b). Altogether, doxycycline ameliorates the loss of GAG contents when cartilage plugs are cultured at 37°C.

### 3.8 Doxycycline protected against reduction of mechanical strength of calf cartilage plugs

**FIGURE 7:**
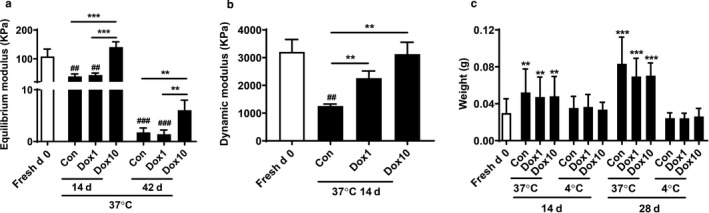
Doxycycline protected against the reduction of mechanical strength of calf cartilage plugs, but had no effect on wet weight of calf cartilage plugs. Calf knee cartilage was punched into plugs with a diameter of 4 mm. The cartilage plugs at the day of slaughter were used as the Fresh d0 group. The plugs were cultured in 48‐well plates at 37°C or 4°C in the presence or absence of doxycycline (1 μg/ml and 10 μg/ml) until 42 days. The wet weights of the cartilage plugs were examined at 14 days and 28 days. Cartilage plugs were removed from their respective mediums at 37°C and 4°C at different time points. Mechanical testing was performed on an Instron Electropuls E1000. Equilibrium modulus was calculated using stress at *t* = 30 min during stress relaxation. Dynamic modulus was calculated using the average of minimum and maximum stresses at the 20th, 30th, and 40th cycle of cyclic loading. (a) Equilibrium modulus (*N* = 3–5). (b) Dynamic Modulus (*N* = 4). (c) Change of wet weights at 14 days and 28 days (*N* = 6). ^##^ *p* < .01, ^###^ *p* < .001 versus Fresh d0. **p < .01, and ***p < .001 for comparison of two groups

As aforementioned, doxycycline treatment increased chondrocyte viability at 37°C. Here we determined the effect of doxycycline on the compressive strength of calf cartilage plugs at 37°C. We found that fresh cartilage plugs had a mean equilibrium modulus of 108.20 kPa at day 0, which was gradually reduced to 1.42 kPa at day 42. Incubation with doxycycline (10 μg/ml) attenuated the reduction of mechanical integrity of cartilage plugs at days 14 and 42, whereas doxycycline at a concentration of 1 μg/ml had no such effects (Figure 7a). Cartilage plugs had a mean dynamic modulus of 3,208 kPa at day 0, which was significantly reduced after being cultured for 14 days (Figure 7b). Incubation with doxycycline (1 and 10 μg/ml) significantly augmented dynamic modulus in a concentration‐dependent manner (Figure 7b). These results indicate that doxycycline incubation is beneficial in maintaining the mechanical integrity of the cartilage.

### 3.9 Doxycycline had no effects on wet weight of calf cartilage plugs during culture

In order to investigate the effect of temperature on the weight of cartilage plugs, the wet weights of the cartilage plugs were examined under the two different temperatures at 14 days and 28 days. Cartilage plugs were removed from their respective mediums at 37°C and 4°C. After removal of excess water by clean Kimwipe tissue paper, the wet weights of the cartilage plugs were measured. We found that throughout the 28‐day preservation period, all groups of cartilage plugs stored at 37°C had noticeably increased in weight as compared to fresh plugs (d0) at both time points (days 14 and 28) (Figure 7c). At both days 14 and 28, cartilage plugs at 37°C had increased wet weights compared to these at 4°C (Figure 7c). Incubation with doxycycline (1 and 10 μg/ml) had no effect on wet weight of cartilage plugs regardless of culture temperatures or time points (Figure 7c). These results demonstrate that weight of cartilage plugs increases during culture at 37°C, which is not affected by doxycycline treatment.

## 4 DISCUSSION

There are two common mediums used to preserve OC grafts: one is serum‐free medium consisting of glucose, salts, and amino acids, and the other is FBS medium containing nutrients and growth factors. FBS media offers the advantage of improved chondrocyte viability, and improved GAG content and histologic appearance at 4°C over 60 days compared to just serum‐free media (Garrity et al., 2012; A. Stoker, Garrity, Hung, Stannard, & Cook, 2012). Despite the prolonged chondrocyte viability when stored in FBS, concerns regarding the variability in solutions and potential for zoonotic disease transmission and contamination associated with FBS made its use less desirable (Pennock et al., 2006). The medium containing DMEM, MEM nonessential amino acid solution, 50 mg/ml L‐ascorbic acid, selenium, insulin, transferrin, and 0.9 mmol/L sodium pyruvate was proven to maintain chondrocyte viability at levels not different from day 0 control through day 56 when storing OC graft at 37°C (Garrity et al., 2012). This may be due to the antioxidant property of L‐ascorbic acid (Chang et al., 2015). Therefore, we chose the same media as Garrity et al. (Garrity et al., 2012) to test if doxycycline can prolong fresh OC grafts’ chondrocyte longevity in this media at 37°C. In agreement with our findings, 37°C storage maintains long‐term (more than 4 weeks) chondrocyte viability, whereas 4°C preservation of OC allografts supports biologically viable cartilage for short duration (2–4 weeks) (Bugbee, Pallante‐Kichura, Gortz, Amiel, & Sah, 2016). The mechanisms underlying these findings were associated with increased apoptosis at 4°C compared to 37°C of storage. Indeed, chondrocyte death during OC allograft storage was regulated by apoptosis (Robertson, Allen, Pennock, Bugbee, & Amiel, 2006).

Donor chondrocyte viability in OC allograft is critical for maintaining the biochemical and biomechanical properties of OC allografts. All successful OC allograft had more than 70% viable chondrocytes at the time of transplantation, and no graft with less than 70% viable chondrocytes has a successful outcome (Cook et al., 2016). In one case report, grafts retrieved after initial implantation of fresh allografts still contained viable chondrocytes after 29 years (Jamali, Hatcher, & You, 2007). Therefore, donor chondrocyte viability is crucial for successful outcomes after OC allograft transplantation. As clinical successful outcomes are associated with OC allografts that have at least 70% chondrocyte viability, current standards at tissue banks require microbiological and serological safety testing of graft specimens that typically lasts up to 14 days. The short practical shelf life of 14 days has narrowed the time window for allograft implantation to merely 15–28 days (Familiari et al., 2018). Thus, we did not detect chondrocyte viability within 14 days of culture. Doxycycline incubation at 10 μg/ml for 28 days and 48 days maintained ~70% viability of chondrocytes, and this may ensure the successful outcome of OC allograft. Nevertheless, further study is required to optimize the doxycycline concentrations and medium constituents to achieve higher chondrocyte viability as well as to maintain matrix integrity and mechanical strength.

Doxycycline has been shown to affect cartilage matrix degradation and the disruption of terminal chondrocyte differentiation (Cole et al., 1994). Doxycycline can also cause decreased MMP‐1 and MMP‐13 collagenase production, reduced mRNA of interleukin (IL)‐1α, IL‐1β, and IL‐6, and upregulated transforming growth factor β3 in the articular chondrocytes from human osteoarthritic cartilage (Familiari et al., 2018; Shlopov et al., 2001). In addition, with doxycycline treatment, there was an increasing trend in levels of chondrogenic inducer (i.e., sox‐9 and transforming growth factor β receptor II), and chondrogenic genes (i.e., aggrecan and collagen II) over chondrogenic culture time (Lee et al., 2013). Our findings further demonstrated that doxycycline incubation increased mitochondrial respiration in human chondrocytes. These results suggest these mechanisms may contribute to the protective role of doxycycline in improving chondrocyte viability.

Previous studies indicate different results in culture time‐dependent GAG content of explants stored at 37°C. One study reported significant decreases in GAG content at 37°C in a serum‐free chondrogenic medium as compared to fresh baseline at 28 days and 42 days (Bian et al., 2008). However, another study found no significant changes in GAG content of cartilage explants stored in a similar serum‐free medium at 37°C between 28 days and 56 days (Garrity et al., 2012). In this study, there was no change in GAG content at 4°C as compared to 37°C after both 28 days and 56 days storage using the same medium (Garrity et al., 2012). Cartilage quality for this storage method was further supported by the findings of tissue GAG and mechanical properties. However, we found that doxycycline at 1 μg/ml exhibited more effectiveness in protecting against GAG reduction as compared to the control group and 10 μg/ml of doxycycline group at both 37°C and 4°C in Safranin O/Fast Green staining, and at 37°C after both 14 days and 28 days in GAG‐DMMB assay. Safranin O/Fast Green staining also showed that GAG content was less at 4°C as compared to that at 37°C at all time points. GAG‐DMMB assay indicated no significant change in GAG content in the control group on 14 days as compared to 28 days at 37°C. Throughout the preservation period, cartilage explants stored at 37°C had noticeably swelling as compared to fresh explants as well as explants at 4°C. This observation is consistent with the fact that at each time point, the plugs at 37°C had significantly greater wet weights than 4°C plugs. GAGs are polysaccharides and have various negatively charged carboxyl and sulfate groups that can maintain water in tissues. GAG chains attract water through hydrogen bonding with the sulfate groups of chondroitin sulfate and keratan sulfate (Miller, Goude, McDevitt, & Temenoff, 2014). Therefore, water loss is expected to accompany decreases in GAG content at 4°C. Previous studies showed that the compressive stiffness of cartilage is mainly determined by GAG and water content, while tensile strength and other short‐term dynamic properties are primarily affected by collagen content. Negatively charged acidic sugar residues and sulfate groups in GAG chains create electrostatic repulsion within the matrix that contributes to the compressive resistance of cartilage (Gandhi & Mancera, 2008; Laasanen et al., 2003; Treppo et al., 2000). Within these storage condition groups, the decreasing trend of equilibrium and dynamic moduli were observed over time.

We noticed that the freshly isolated primary chondrocytes displayed a typical round shape, however, passaged chondrocytes in culture showed features of a dedifferentiated phenotype, and the cells displayed spindled‐shaped morphology. Whether these cells change chondrocyte phenotype during culture remains to be investigated, despite they still expressing collagen II. Due to insufficient human cartilages, we are unable to study the chondrocyte viability in human cartilage plugs between 4°C and 37°C culture. Further study is required to determine whether doxycycline exhibits differential effects on chondrocyte viability in fresh OC grafts among adolescent, young and adult cartilages. As all experiments were conducted in vitro, there are certainly inherent limitations with this study. Although chondrocyte viability, mechanical properties, and immunohistochemistry staining were performed to measure cartilage explant quality, none of these are direct measures of in vivo functions.

In conclusion, preservation at 37°C is beneficial for maintaining chondrocyte viability in cartilage plugs compared to 4°C. Incubation of doxycycline protects against reduced chondrocyte viability and impaired mechanical properties in cartilage plugs, which is associated with reduced apoptosis and increased mitochondrial respiration. This study provides a potential approach of using doxycycline at 37°C to preserve chondrocyte viability in fresh OC grafts for treatment of articular cartilage lesions.

## CONFLICT OF INTEREST

Brett D. Owens is a consultant of MTF/Conmed, Mitek, Vericel, MIACH, receives royalties from Conmed, and holds stock options in Vivorte. Other authors have no conflict of interest to disclose.

## AUTHOR CONTRIBUTIONS

Conception and Design: BDO; Data acquisition and analysis: LY, BV, HY; Data Interpretation: LY, BV, HY; Drafting the manuscript: LY; Revising the manuscript: LY, HY, BDO.

## ETHICAL STATEMENT

The protocol for processing human OC grafts (Project#: 1240330‐2) was approved by the IRB committee at the Rhode Island Hospital. The protocol for processing calf articular cartilage was approved by the Rhode Island Hospital IACUC (Project#: 1319440‐1).

## Data Availability

Data are available from the authors upon reasonable request.
